# *Tbx18* Regulates the Differentiation of Periductal Smooth Muscle Stroma and the Maintenance of Epithelial Integrity in the Prostate

**DOI:** 10.1371/journal.pone.0154413

**Published:** 2016-04-27

**Authors:** C. Chase Bolt, Soumya Negi, Nuno Guimarães-Camboa, Huimin Zhang, Joseph M. Troy, Xiaochen Lu, Andreas Kispert, Sylvia M. Evans, Lisa Stubbs

**Affiliations:** 1 Department of Cell and Developmental Biology, University of Illinois at Urbana-Champaign, Urbana, Illinois, United States of America, 61801; 2 Institute for Genomic Biology, University of Illinois at Urbana-Champaign, Urbana, Illinois, United States of America, 61801; 3 Skaggs School of Pharmacy, Department of Medicine, and Department of Pharmacology, University of California San Diego, La Jolla, CA, United States of America, 92037; 4 Illinois Informatics Institute, University of Illinois at Urbana-Champaign, Urbana, Illinois, United States of America, 61801; 5 Institut für Molekularbiologie, OE5250, Medizinische Hochschule Hannover, Carl-Neuberg-Str. 1, D-30625 Hannover, Germany; Stanford University School of Medicine, UNITED STATES

## Abstract

The T-box transcription factor TBX18 is essential to mesenchymal cell differentiation in several tissues and *Tbx18* loss-of-function results in dramatic organ malformations and perinatal lethality. Here we demonstrate for the first time that *Tbx18* is required for the normal development of periductal smooth muscle stromal cells in prostate, particularly in the anterior lobe, with a clear impact on prostate health in adult mice. Prostate abnormalities are only subtly apparent in *Tbx18* mutants at birth; to examine postnatal prostate development we utilized a relatively long-lived hypomorphic mutant and a novel conditional *Tbx18* allele. Similar to the ureter, cells that fail to express *Tbx18* do not condense normally into smooth muscle cells of the periductal prostatic stroma. However, in contrast to ureter, the periductal stromal cells in mutant prostate assume a hypertrophic, myofibroblastic state and the adjacent epithelium becomes grossly disorganized. To identify molecular events preceding the onset of this pathology, we compared gene expression in the urogenital sinus (UGS), from which the prostate develops, in *Tbx18*-null and wild type littermates at two embryonic stages. Genes that regulate cell proliferation, smooth muscle differentiation, prostate epithelium development, and inflammatory response were significantly dysregulated in the mutant urogenital sinus around the time that *Tbx18* is first expressed in the wild type UGS, suggesting a direct role in regulating those genes. Together, these results argue that *Tbx18* is essential to the differentiation and maintenance of the prostate periurethral mesenchyme and that it indirectly regulates epithelial differentiation through control of stromal-epithelial signaling.

## Introduction

During middle and late gestation of the mouse, the T-box transcription factor (TF) TBX18 is expressed in a population of mesenchymal cells in the lower embryonic abdomen. These cells contribute to the stromal layer of nearly every organ in the urogenital system but with differing affects in each of them [1]. In the ureter, *Tbx18* is essential to the formation of a coordinated smooth muscle layer that can conduct urine from the kidney to the bladder. Beginning at embryonic day 11.5 (E11.5) *Tbx18*-expressing mesenchymal cells begin to coalesce around the nascent ureter epithelial duct [2]. Secreted SHH and WNT signals from the ureter epithelium maintain the proliferation and eventual differentiation of these *Tbx18*-positive condensing mesenchymal cells [3–5]. However, in the absence of *Tbx18*, the mesenchymal cells fail to respond to the epithelial signals and subsequently retire to a fibrocytic fate [2]. Consequently, a still unknown signal acting downstream of *Tbx18* in the mesenchyme, which normally reciprocates the proliferation signal to the ureter epithelium, fails to be activated. Due to the loss of these interdependent signaling mechanisms, neither the ureter epithelium nor the stroma proliferate sufficiently resulting in a ureter of reduced length, thickness, and elasticity. The consequent fluid build-up leads to a grotesque enlargement of both the ureters and kidneys in *Tbx18* mutants [2,6].

Toward the posterior end of the urogenital system, the prostate is an organ essential to male fertility that arises developmentally from the urogenital sinus (UGS) [7]. Beginning at E16.5, the urogenital sinus mesenchyme (UGS-M) begins differentiating under the influence of testicular androgens and then induces the adjacent urogenital sinus epithelium (UGS-E) towards a path of prostate committal [8,9]. The naïve UGS-E, responding to signals emanating from the UGS-M, begins to invade the adjacent undifferentiated mesenchyme beginning around E17.5 [10–13]. As the epithelial buds extend into and beyond the UGS-M, mesenchymal cells condense around the buds producing the early rudiments of the prostate stromal layer, comprised mostly of smooth muscle cells and fibroblasts [14]. Paracrine signals including *Wnt* [15,16], *Notch* [17], *Bmp* [12,18], *Tgfb* [19,20] and *Shh* [21,22] play critical roles in this process of prostate bud induction and differentiation, each with regional expression patterns and differential affects on the formation of the various lobes. Furthermore, abnormal expression of these factors and their downstream receptor pathways are indicative, and sometimes directly causative, of lobe specific prostate pathologies such as fibrosis and neoplasias [19,23–25].

In the adult mouse prostate, the prostatic lobe stroma adjacent to the urethra consists of a core of smooth muscle cells (SMCs) with a few VIM+ fibroblasts positioned at the lobe perimeter [26,27]. The well-organized smooth muscle bundles produce large amounts of *Tgfb1*, which inhibits proliferation of the adjacent epithelial stem cell population; the loss of *Tgfb1* signaling induces epithelial neoplasias [28,29]. Paradoxically, in prostatic models of “reactive stroma”, SMCs and fibroblasts in the stroma experience up-regulation of *Tgfb1* production resulting in progressive induction of the stroma towards a diseased myofibroblast phenotype [20,30]. These myofibroblasts, distinguished by co-expression of SMA and *Vimentin* (VIM), exhibit elevated production of ECM components (collagens), Transforming growth factors (*Tgfb1*), and matrix remodeling enzymes (Serpines and Cathepsins) to create a growth-promoting microenvironment [31]. As a consequence of these changes in the stromal microenvironment, the adjacent epithelium experiences hyper-proliferation and can become invasive [17,20,29,30]. A common outcome from this situation is the formation of Prostatic Intraepithelial Neoplasia (PIN) [32].

A number of studies have indicated that *Tbx18* contributes to urogenital structures aside from the ureter and bladder [1,33,34]. However, since *Tbx18*-null animals die perinatally [35], phenotypic effects in the postnatally developing prostate have been impossible to discern. We recently reported a novel hypomorphic *cis*-regulatory mutation of *Tbx18*, called 12Gso [6], and the relative longevity of these animals offered the opportunity to examine *Tbx18-*related postnatal phenotypes. Using this mutant in combination with a novel *Tbx18* conditional allele, here we demonstrate that *Tbx18* regulates differentiation of a SMC subpopulation that contribute to the periductal prostate stroma, particularly within the mouse anterior lobe. In contrast to the ureter, which displays reduced stromal and epithelial thickness in mutant animals, periductal stroma proximal to the urethra is hypertrophied in *Tbx18* mutant adult prostates, composed primarily of enlarged and disoriented SMCs and myofibroblasts. Furthermore, prostatic epithelial cells surrounded by this abnormal stroma are significantly disorganized by early adulthood, and the abnormal ductal regions also contain unusually large numbers of disorganized, Vimentin-positive cells. Together, our data indicate an important role for *Tbx18* in regulating the reciprocal epithelial-stromal signaling from the earliest stages of prostate development, with implications for human prostate disease.

## Methods

### Histopathology and Immunohistochemistry

Tissues were dissected at the appropriate gestational or postnatal stage. Tissues were fixed in 4% PFA at 4°C, dehydrated, and embedded in paraffin wax for sectioning. 4–6μm sections were used in all experiments. TBX18-2 antibody is used at 1:800 in normal goat serum blocking solution and incubated overnight at 4°C. Following washes, a Goat anti-Rabbit:HRP (Jackson ImmunoResearch 111-035-003 1:500) secondary antibody was applied for 1hr at room temperature. Sections were covered in TSA-Rhodamine for 6 minutes then washed and counter stained with Hoecsht 33342. Commercial antibodies are VIM (Abcam ab92547 1:500), CK8/CK18 (ab53280 1:100), CK5 (ab24647 1:750), and SMA (ab7817 1:75, ab5694 1:1000) with secondary antibodies (from Thermo-Fisher Scientific), Goat anti-Rabbit IgG, Alexa Fluor 488, A-11008 1:200 and Goat anti-Mouse IgG, Alexa Fluor 568, A-11004 1:200 and Alexa Fluor 647, A-31573, 1:200.

### RT-qPCR

Total RNA was collected by homogenizing tissues in Invitrogen Trizol reagent. RNA was DNase I treated with NEB DNase I, and cleaned on Zymo Research RNA Clean & Concentrate 25 columns. NEB M-MULV reverse transcriptase was used to generate cDNA. Primer sequences were obtained from PrimerBank [36] and validated prior to use. Primer sequences are listed in S3 Table. An Applied *Biosciences* QuantStudio Flex 6 thermocycler was used for amplification. The delta-delta-Ct method was employed to establish quantity differences.

### RNA-Seq

Whole urogenital sinuses were dissected from male mouse embryos at embryonic day E16.5 and E18.5. Tissues were homogenized in Trizol and RNA was isolated. RNA was treated with NEB DNase I, then analyzed on a Bioanalyzer for quality. RNA-Seq libraries were prepared with KAPA Biosystems KAPA Stranded mRNA-Seq Kit for Illumina Platform (KR0960—V2.14). Single-end reads were performed on Illumina Hi-Seq 2000 sequencers. Single stranded sequencing reads were processed with Tophat 2.1.0 (using Bowtie 2.1.0 and Samtools 0.1.19) with all Tophat options at default. Cufflinks 2.2.1 was used to determine differential expression. Results were filtered to consider only those genes with at least 1 FPKM in either the mutant or control samples for the final selection of differentially expressed genes. Datasets reported in this study have been deposited into the GEO database (http://www.ncbi.nlm.nih.gov/geo/) under accession number GSE80083.

### Mouse breeding

All mouse lines used were bred onto B6C3-F1 hybrid background because genetic background appears to influence *Tbx18* phenotype severity [6]. All animal work described in this study, including euthanasia, was carried out in strict accordance with the recommendations in the Guide for the Care and Use of Laboratory Animals of the National Institutes of Health. The protocol was approved by the Institutional Animal Care and Use Committee of the University of Illinois (Animal Assurance Number: A3118-01; approved IACUC protocol number 15245)

### Generation of the *Tbx18*^*Flox*^ allele

The conditional *Tbx18* allele was generated by inserting LoxP sites flanking the second exon of the endogenous *Tbx18* gene. In crosses with mice carrying the epibliastic *Meox2-*Cre knockin allele [B6.129S4-*Meox2*^*tm1(cre)Sor*^/J [37]; obtained from the Jackson Laboratory, Bar Harbor, ME] homozygous *Tbx18* ^*flox/flox*^ mice displayed phenotypes identical to those previously published for global *Tbx18*-null mice, confirming the efficient ablation of *Tbx18*. A more detailed description of phenotypes associated with this allele will be forthcoming (Guimarães-Camboa et al., manuscript in preparation).

## Results

### *Tbx18* is expressed in the early urogenital sinus mesenchyme

Previous reports of the *Tbx18* expression pattern in the urogenital system have not included analysis of the urogenital sinus or prostate [1,2]. However, the prostate develops at the base of the bladder in near proximity to the ureter, and the obvious parallels between the development of these two structures prompted a careful re-evaluation.

**Fig 1:**
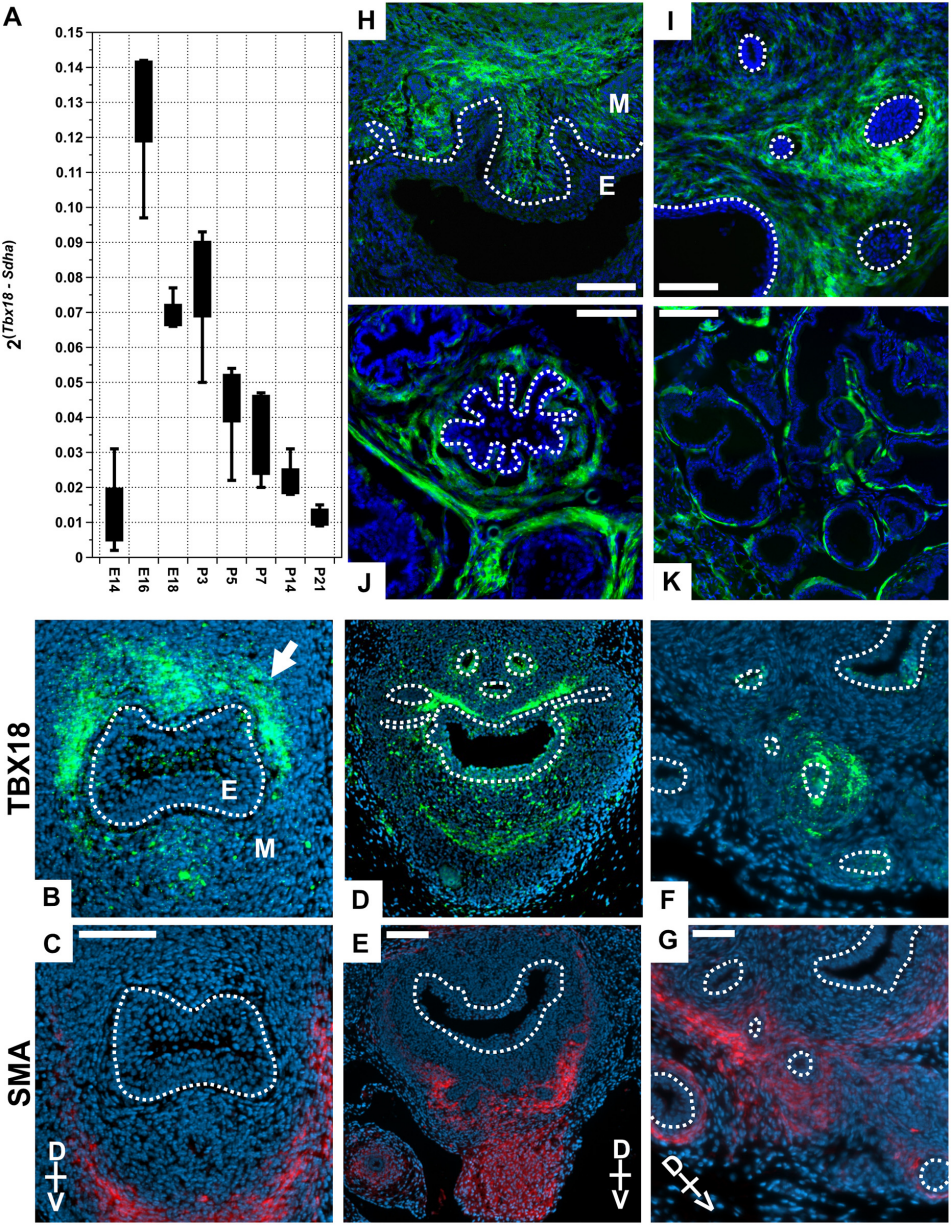
*Tbx18* expression in the urogenital sinus mesenchyme. (A) Quantitative reverse transcription PCR detects *Tbx18* mRNA as early as E14.5, with expression peaking around embryonic day 16.5 (for each stage n ≥ 4). (B, D, F) TBX18 immunohistochemistry shows TBX18 is expressed in the dorsal aspect of the UGS in the region of the forming anterior prostate buds at E16.5 (B), P0 (D), and P3 (F). (C, E, G) Smooth Muscle Actin (SMA) IHC on sections adjacent to TBX18 stains showing the proximity of these two expression domains; TBX18 is detected in only a small subset of SMA-positive cells. (H-K) Lineage tracing analysis of *Tbx18* expressing cells using the *Gt(ROSA)26Sor*^*tm4(ACTB-tdTomato*,*-EGFP)Luo*^/J reporter. Green cells express or are descended from *Tbx18* expressing cells (in the images presented here, the *Tomato* signal was excluded for clarity). (H-I) P0. (J-K) P35 sections show *Tbx18* descendants primarily occupy the anterior prostate stroma (J), and dorsal prostate stroma (K). M is Mesenchyme, **and E is Epithelium. Scale bars are 100μm B and C, 50μm in D-K.**

We set out to determine the quantity and location of native *Tbx18* expression in prostate through the late stages of gestation and the postnatal days through puberty (Fig 1). First we used quantitative reverse transcript PCR (RT-qPCR) to detect *Tbx18* mRNA in the UGS beginning at E14.5, before prostate development initiates, through gestation and the first few days after birth. *Tbx18* mRNA was barely detectable at E14.5, rose dramatically by E16.5, and dropped slowly throughout the first week postnatal. Shortly after P3 and continuing over the first two weeks of postnatal development, *Tbx18* mRNA levels dropped towards the limits of qPCR detection (Fig 1A).

To provide spatial resolution of TBX18 protein localization, we used immunohistochemistry (IHC) with a tested TBX18 antibody [6] in sectioned E16.5 to P3 animals. At E16.5, TBX18 staining was observed primarily on the dorsal aspect of the UGS-M, proximal to the epithelium in regions from which the dorsal and anterior prostate buds will eventually emerge (Fig 1B). At P0, TBX18 was also detected adjacent to the dorsal UGS-E with staining around the proximal regions of the epithelial prostate buds (Fig 1D) and a few isolated cells ventral to the UGS-E. By P3, TBX18 was detected in clusters of cells in the UGS-M proximal to the prostate epithelial buds (Fig 1F). Due to the known role of *Tbx18* in smooth muscle formation, we also examined the relationship between TBX18 expression and smooth muscle formation by evaluating alpha-Smooth Muscle Actin (SMA) antibody staining on sections adjacent to those stained for TBX18. Consistent with previous reports of TBX18 and SMA expression, the expression of these two proteins were mostly spatially and temporally separated [2]. SMA staining was frequently observed in cells in the vicinity of TBX18-positive cells, but the two proteins were largely detected in non-overlapping domains (Fig 1C, 1E and 1G). Together these analyses indicated that *Tbx18* UGS expression is primarily limited to late gestation and early postnatal stages. The position of the TBX18*+* cells adjacent to nascent prostate buds suggested they may contribute to the adult prostate stroma.

We used a lineage-tracing system to identify the adult prostate regions and cell types to which TBX18+ embryonic cells contribute, crossing mice carrying a *Cre* recombinase knock-in to the *Tbx18* locus [38] with the *Gt(ROSA)26Sor*^*tm4(ACTB-tdTomato*,*-EGFP)Luo*^/J (mT/mG) reporter line. In these animals, only *Cre-*expressing cells and their descendants will express eGFP [39]. We observed GFP expression, indicating the presence of *Tbx18-*driven *Cre* activity, first at E16.5 in the UGS-M (not shown) and at P0 with the same patterns as that detected in IHC (Fig 1H and 1I). In prostates of 5 week-old animals, we observed GFP expression in the periductal stromal smooth muscle layer of the anterior and the dorsolateral lobes confirming that *Tbx18*-expressing cells contribute significantly to prostate stromal SMCs (Fig 1J and 1K).

### Subtle abnormalities in the nascent prostate of *Tbx18* mutant mice

*Tbx18* expression in the UGS during embryonic and postnatal stages suggested that *Tbx18* might play a role in prostate development. However, most prostate development occurs postnatally precluding the use of *Tbx18*-null mutations to identify later prostate phenotypes. Since ureter expression and development are disrupted in 12Gso mice [6], we reasoned that this hypomorphic allele might be associated with *Tbx18* gene expression differences and phenotypes in the prostate as well.

We first evaluated the affects of *Tbx18* abrogation by comparing UGS morphology in *Tbx18*^*GFP/GFP*^ null mutants [38], hypomorphic 12Gso/*Tbx18*^*LacZ*^ [6,35] compound heterozygotes, and wild type littermates at two prenatal stages: E16.5, before the time that prostate budding begins, and E18.5, after the process has initiated. At both of these prenatal stages we observed no morphological differences between animals of the three genotypes (data not shown).

**Fig 2:**
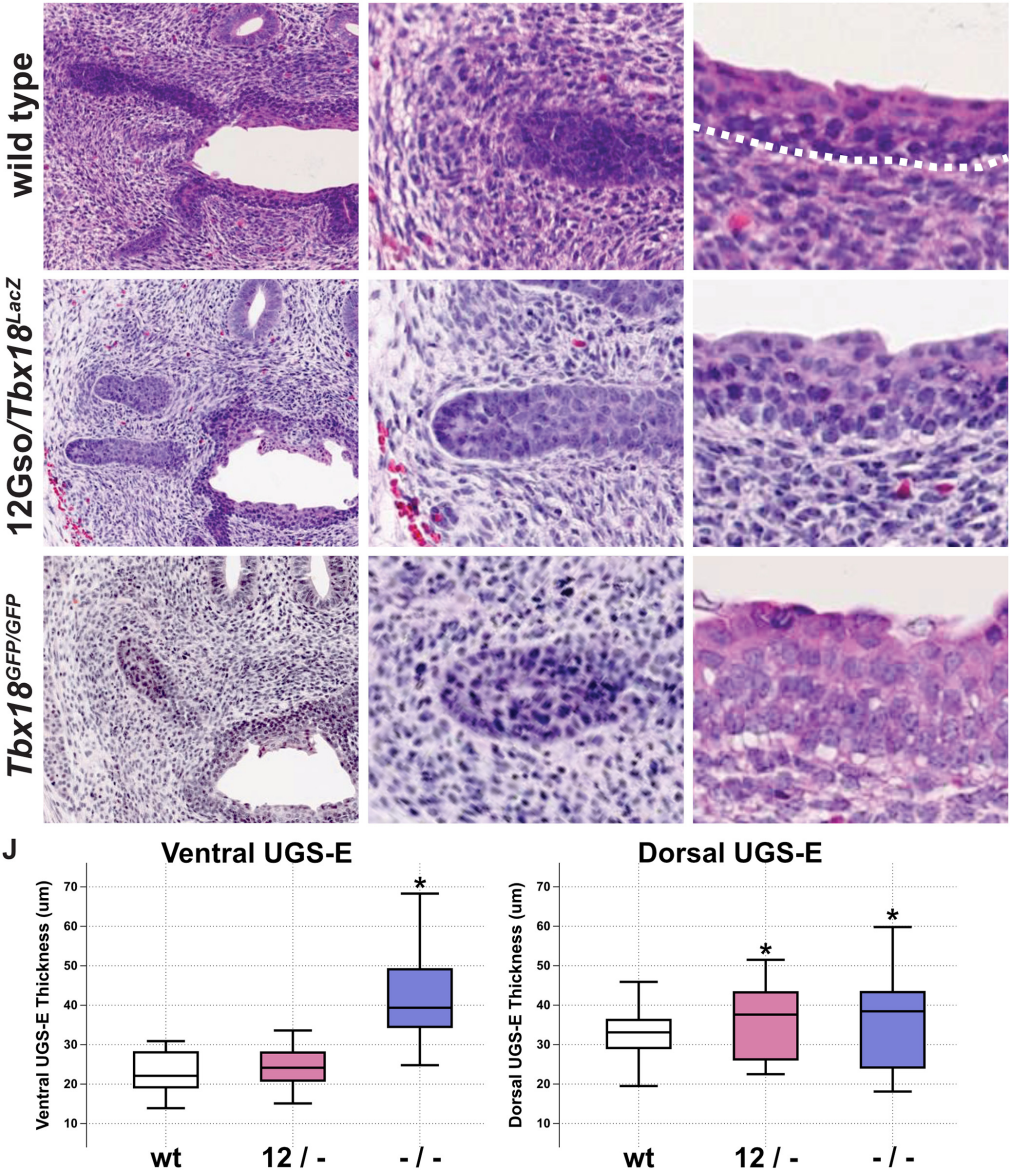
*Tbx18* LOF phenotype in P0 UGS. (A-I) H&E stains of P0 urogenital sinuses. (A-C) Wild type histology shows high cell density in the mesenchyme surrounding the epithelial prostate buds (A, B), and urethral epithelium is composed of 4–6 cell layers with a smooth apical surface (C). (D-F) 12Gso/*Tbx18*^*LacZ*^ compound heterozygotes present an intermediate phenotype in the UGS mesenchyme and the urethral epithelium. (G-I) *Tbx18*^*GFP/GFP*^ mutants have very low mesenchymal cell density surrounding epithelial prostate buds. The urethral epithelium in these mutants is increased in thickness with larger cell volumes (compare arrows in C, F, and I). (J) Measurements of the epithelial thickness in the urethral epithelium. The epithelium on the dorsal side is significantly increased in thickness compared to wild type littermates.

However, at P0, we observed two subtle but consistent changes (Fig 2). We sectioned through the entire abdominal region including the developing prostate in multiple animals (n = 7 of each genotype) and stained slides at regular intervals to compare morphology in a large sampling of sectioning planes. The differences between mutant and wild type prostates at this stage were subtle but consistently observed throughout the organs. First, in animals of both mutant genotypes, the apical surface of the urethral epithelium displayed increased convolutions and irregularities compared to wild type (*Tbx18*^*+/+*^) littermate controls (Fig 2A, 2D and 2G). In the *Tbx18-*null animals, we also noticed that epithelial cells displayed increased cell size and nuclear volume (arrows in Fig 2C, 2F and 2I). In addition, the epithelium in both mutant genotypes was relatively thicker, with additional layers protruding into the lumen from the dorsal and ventral sides, than was observed in wild type littermate controls (Fig 2C, 2F and 2I). We quantified the differences by measuring the thickness of the urethral epithelium (luminal surface to basement membrane) at ten positions on both the dorsal and ventral aspect in wild type and *Tbx18* mutants, and confirmed that the ventral urethral epithelium was increased in *Tbx18*^*GFP/GFP*^ mutants, while the dorsal epithelium thickness was increased in both mutants (Fig 2J). We also observed that the characteristic pattern of higher UGS-M cell density around the forming prostate buds of wild type mice was absent in *Tbx18*^*GFP/GFP*^ mutant animals and reduced in 12Gso/*Tbx18*^*LacZ*^ compared to wild type litter mates (Fig 2B, 2E and 2H).

### Loss of *Tbx18* affects the structure and integrity of the anterior prostate lobe

**Fig 3:**
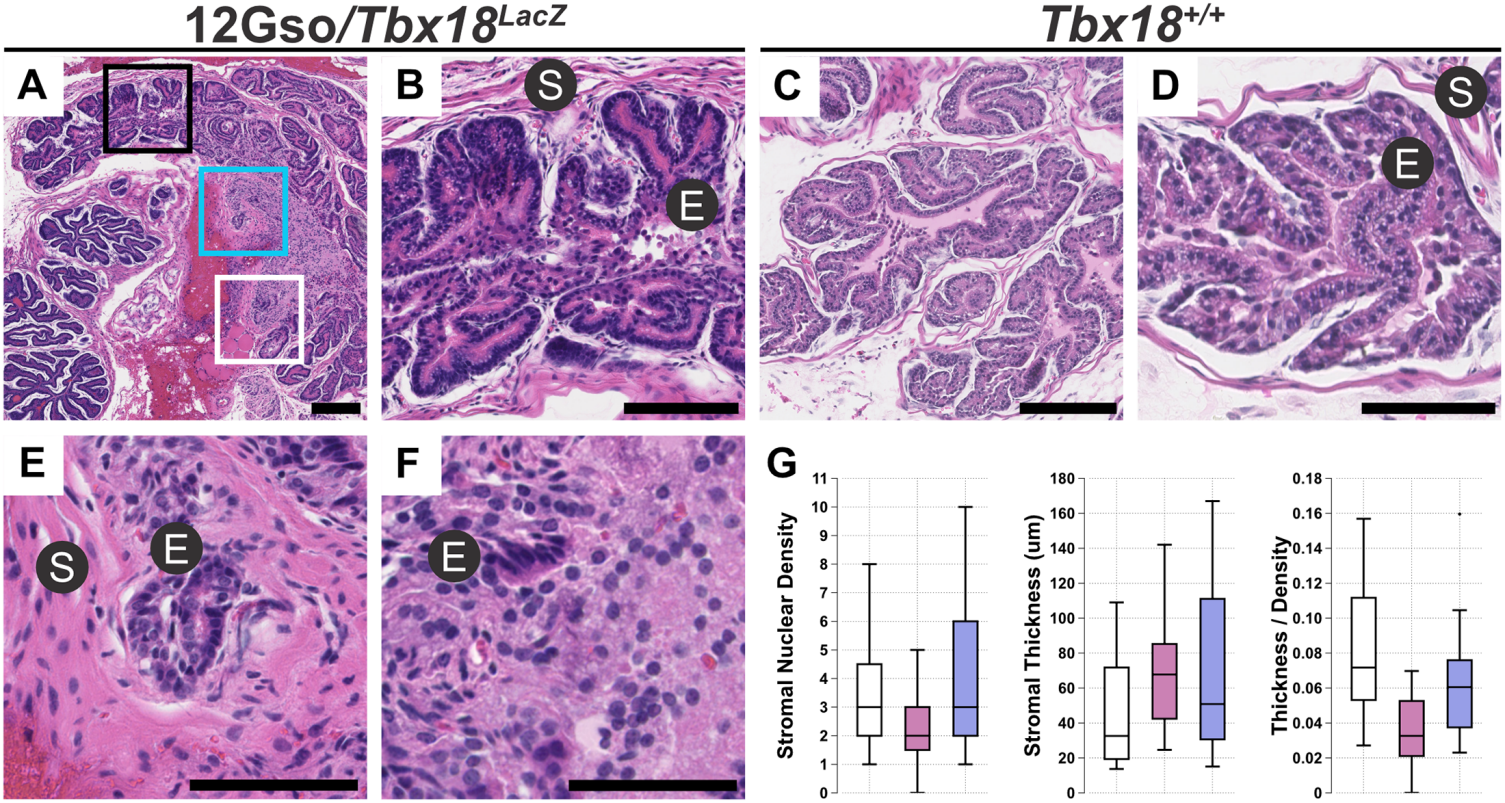
*Tbx18* LOF in 12Gso/*Tbx18*^*LacZ*^ mutants leads to prostate abnormalities in adults. The anterior prostates of five week-old 12Gso/*Tbx18*^*LacZ*^ mutants include stromal hypertrophy and epithelial disorganization, as revealed by Hematoxylin and Eosin (H&E) stains. Sectioned anterior prostates of 12Gso/*Tbx18*^*LacZ*^ mutants (panel A, with areas highlighted in black, blue, and white boxes shown at high resolution in B, E, F respectively) include distal regions with relatively normal morphology (B) indistinguishable from that of wild type littermates (C, D). However, the same sections of mutant prostates show significant stromal hypertrophy (E) and epithelial disorganization and lack a clear boundary between stromal and epithelial compartments (F) in regions proximal to the urethra. (G) Measurement of stromal thickness and numbers of nuclei within the stromal regions confirm that both measurements are significantly different in mutant proximal anterior prostates than in wild type littermate controls. S is stroma; E is epithelium. Scale bars correspond to 100μm in all panels.

Most of the prostate develops postnatally, with a significant amount of growth and differentiation during adolescent and pubertal stages up to five weeks after birth [7]. We therefore reasoned that more striking morphological differences might be detected in mutant animals at 5 weeks. Although 12Gso*/Tbx18*^*LacZ*^ compound heterozygotes only occasionally survive to adulthood, we did observe a striking phenotype in two 5 week-old males we examined (Fig 3). Subtle abnormalities were detected in the dorsolateral lobes, but the most striking phenotypes were limited to the anterior prostate.

When the anterior prostates of these mutants were sectioned along the axis proximal-distal to the urethra, we observed a very similar phenotype in both animals. Specifically, the regions of the anterior ducts located distal of the urethra displayed overtly normal structure, including organized epithelial folds encased by thin layer of condensed smooth muscle stromal cells (Fig 3A and 3B). We could discern no obvious difference between these distal regions of mutant anterior prostates and those of wild type littermate controls (Fig 3C and 3D). However, in the more proximal regions of the same ducts, the mutant anterior prostates were significantly different from those in wild type animals in two respects. First, we noted a marked increase in the thickness of the stromal smooth muscle layer, containing cells that were significantly increased overall in size and misshapen, compared to age-matched wild type prostates (Fig 3A, 3E, 3C and 3D). Second, we observed that epithelium directly adjacent to the disorganized stroma in the mutants lacked the typical well-organized morphology and cell layering seen in comparable wild type sections. Rather, cells in the epithelial compartments of these proximal regions were disorganized and appeared to be loosely associated in the mutant mice (arrowhead in Fig 3E and 3F).

To quantify cell number and cell size we measured the stromal thickness (the distance from the epithelial basement membrane to the distal stromal periphery) at twenty positions around the anterior prostate lobes and counted the number of nuclei along the same linear path of measurement (Fig 3G). We found that the median stromal width was significantly larger in 12Gso/*Tbx18*^*LacZ*^ mutants than in wild type littermates on average: 32μm in wild type, versus 67μm and 50.8μm for 12Gso/*Tbx18*^*LacZ*^ mutant prostates respectively. By measuring the ratio of the number of nuclei per stromal width, we observed a decrease in the number of nuclei per width in the prostate stroma of 12Gso/*Tbx18*^*LacZ*^ mutants (Fig 3G), supporting a hypertrophy model. Thus it appears the abnormal stromal thickness is due to cells occupying the periductal space normally reserved for stromal SMC were enlarged and lacked the condensed, parallel organization observed in wild type mice. In these morphological characteristics, the abnormal stromal cells in *Tbx18* mutant prostates resemble myofibroblasts, which are frequently associated with prostate pathologies [30,40,41].

### Conditional abrogation of *Tbx18* in the prostate

**Fig 4:**
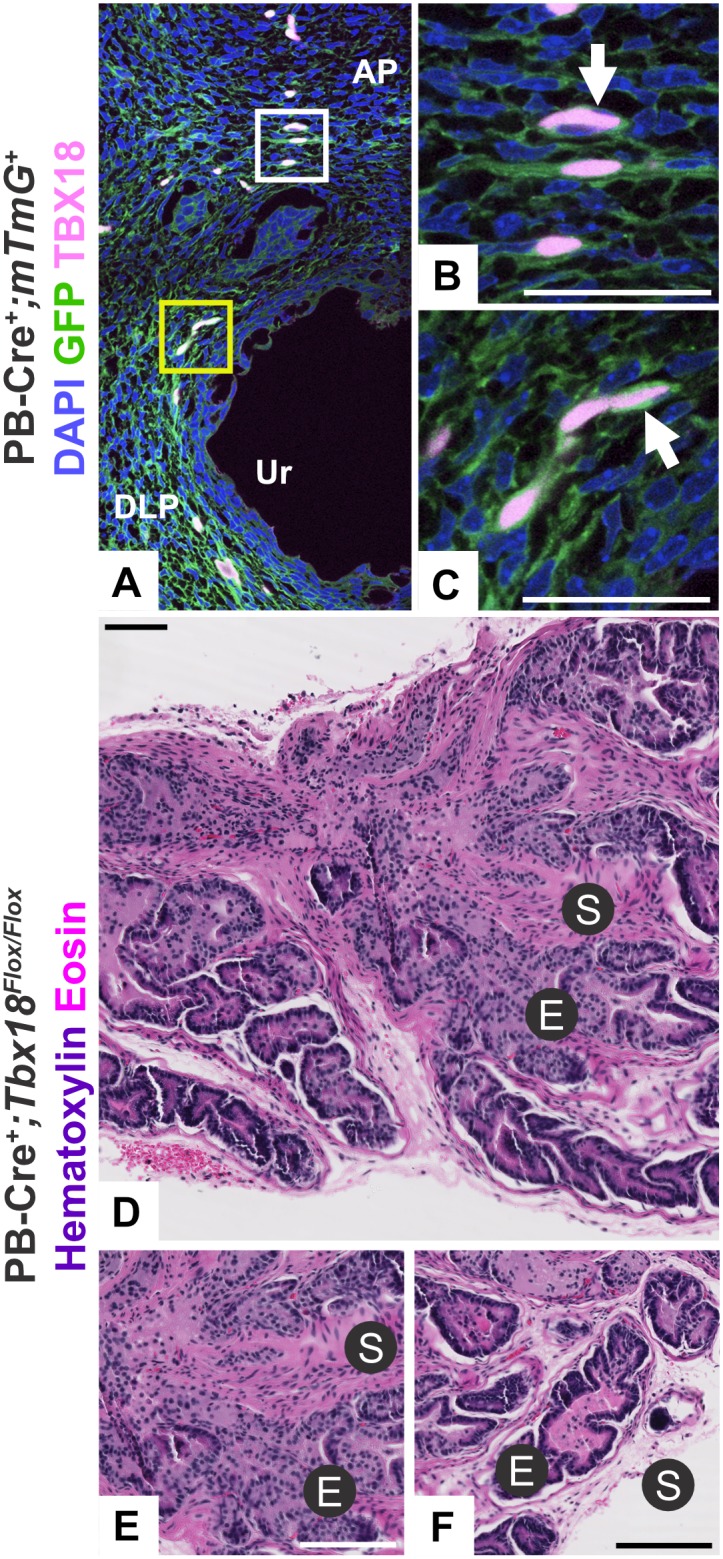
Conditional ablation of *Tbx18* in the prostate. (A) The PB-Cre4 transgene is active at P0 in the prostatic mesenchyme as shown by the mT/mG reporter line, and (B, C) cells with *Cre* expression co-stain with TBX18 around the anterior buds. (D-F) PB-Cre^+^; *Tbx18*^*Flox/Flox*^ animals display similar pathology to the 12Gso/*Tbx18*^*Lacz*^ animal at P35, with (E) urethra-proximal regions of stromal hypertrophy and epithelial disorganization and (F) distal regions of the same ducts displaying normal pathology. Scale bars correspond to 100μm in figures (A, D-F) and 50μm in figure (B,C). AP is anterior prostate mesenchyme. DLP is dorsolateral prostate mesenchyme. Ur is urethra.

The survival of 12Gso/*Tbx18*^*LacZ*^ animals to adulthood was sufficiently rare to impede efficient further investigation. To overcome this problem, we obtained a conditional allele of *Tbx18* (*Tbx18*^*flox*^). To remove *Tbx18* function only in the developing prostate, we required a tissue specific *Cre* that would not interfere with *Tbx18* function in other tissues that have previously been associated with loss-of-function lethality. We therefore chose to abrogate *Tbx18* in the prostate utilizing a well-tested transgenic mouse line expressing *Cre*-recombinase from a transgenic rat *Probasin* gene promoter (PB-Cre4) [42] (Fig 4).

PB-Cre4 is expressed at highest levels in the postnatal mouse prostate epithelium and is most commonly used to study epithelial development. However, this transgene is also expressed within the mesenchyme adjacent to the dorsal and anterior lobes, with activity in the mesenchyme reported as early as P1 [17,43]. We confirmed the mesenchymal expression of PB-Cre4 and the overlap between *Cre* and TBX18 expression in prostate mesenchyme using mT/mG reporter strain co-stained with TBX18 antibody. In PB-Cre4^*+*^; mT/mG^*+*^ mice, we observed *Cre* activity in UGS-M cells surrounding the emerging prostate buds, as early as P0 (Fig 4A). In sections co-stained with the TBX18 antibody we confirmed that *Tbx18* and PB-Cre4 are co-expressed in a subset of the same stromal cells around the emerging prostate buds at this stage, with TBX18 protein (stained with far red and shown in pink) localized clearly in the nucleus of cells that were also expressing membrane-targeted GFP (green in Fig 4A–4C).

To evaluate the conditional loss of *Tbx18* in these cells, we produced PB-Cre4^+^, *Tbx18*^*flox/flox*^ animals and collected prostates at 5.5–6.5 weeks of age (n = 6 animals). The phenotype we observed in these animals was highly consistent with that of the 12Gso/*Tbx18*^*LacZ*^ phenotype, characterized by patches of tissue with a thickened prostate stroma surrounding a highly disorganized epithelium. As in 12Gso/*Tbx18*^*LacZ*^ prostates, this phenotype was focused primarily in the anterior prostate lobes and in regions proximal to the urethra, with obviously abnormal ducts adjoined to normally structured acini in the same tissue sections (Fig 4D–4F). Therefore, the inheritance of the 12Gso/*Tbx18*^*LacZ*^ genotype and deletion of *Tbx18* in PB-Cre4-expressing cells yield very similar effects on prostate development.

### Immunohistochemical markers confirm the presence of myofibroblasts and loss of epithelial cell identity in mutant prostates

**Fig 5:**
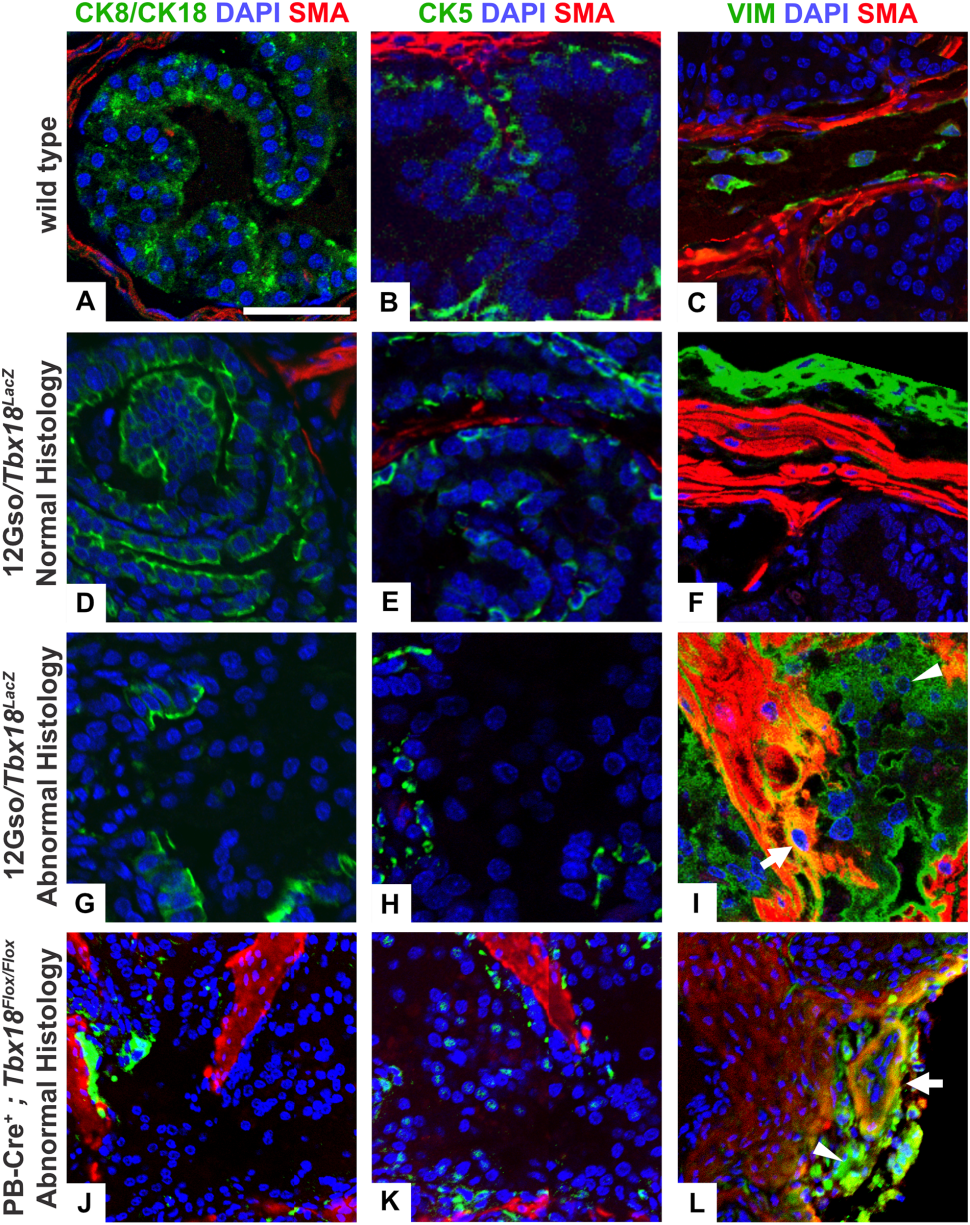
Immunohistochemical analysis with stromal and epithelial markers. We used immunohistochemistry to examine the distribution of stromal and epithelial markers in sections of 5 week-old (A-C) wild-type, (D-I) 12Gso/*Tbx18*^*LacZ*^, and (J-L) PB-Cre4^*+*^; *Tbx18*^*flox/flox*^ anterior prostates. IHC was carried out on slides adjacent to those imaged in Figs 3 and 4 and include the same regions of the tissue. (A) Luminal epithelial cells stained with an antibody detecting Cytokeratins 8 and 18 (CK8/CK18, green) form a continuous layer on the apical surface of the epithelial compartment in wild type prostate and (D) in distal ductal regions with normal pathology in the 12Gso/*Tbx18*^*LacZ*^ compound heterozygotes. (B, E) Basal epithelial cells stained with Cytokeratin 5 (CK5, green) also stain similarly in the wild type and distal regions of the mutant ducts, forming a discontinuous layer between the apical and basement membrane. (G, H, J, K) In contrast, CK8/CK18 and CK5 staining is significantly reduced in the epithelial compartments of the proximal anterior prostate regions in both 12Gso/*Tbx18*^*LacZ*^ (G, H) and PB-Cre4^+^; *Tbx18*^*flox/flox*^ mice (J, K). SMA (red) was co-stained with the Cytokeratin markers to define the smooth muscle layer of the stroma in each section. (C, F) Smooth muscle cells, identified with SMA (red), and fibroblasts, stained with the VIM antibody (green) form apposed but separate layers around the epithelial compartments in wild type animals and in distal regions of normal histology in the anterior prostate of 12Gso/*Tbx18*^*LacZ*^ mice. (I, L) However, in both types of *Tbx18* mutants we found cells positive for both the VIM and SMA markers (arrow in Fig I, L), indicative of myofibroblasts. In addition, compared to wild type littermates (C), the mutant anterior lobes contained larger numbers of VIM+ cells (green; arrowheads in I, L). Scale bars correspond to 100μm.

To further characterize these abnormal prostate phenotypes, we stained mutant and control prostate sections for expression of diagnostic molecular markers. We used slides directly adjacent to those highlighted in Figs 3 and 4 for immunohistochemical staining, comparing staining patterns from both types of *Tbx18* mutants to wild type age-matched controls, with very similar results (Fig 5). First we asked whether epithelial cell-type balance was being maintained by examining mutant and wild type sections with cytokeratin markers of basal or luminal epithelial cell identity (CK5 and CK8/CK18, respectively) [44]; these slides were co-stained with the SMA antibody to delineate stromal and epithelial layers clearly. Next, we examined prostate sections adjacent to those stained with CK markers, after co-staining with Vimentin (VIM) and SMA. These proteins separately mark fibroblasts and smooth muscle cells, respectively, but when expressed together are characteristic of the myofibroblastic phenotype [30].

*Tbx18*-mutant anterior lobes contained stromal smooth muscle cells and epithelium at the distal portions of the lobes that matched the organization and staining patterns of wild type lobes at equivalent positions (Fig 5A–5C). In these normally structured regions, a layer of flattened, SMA-positive cells surrounded an organized epithelium. In the epithelium, a basal layer marked by CK5, and a luminal layer of CK8/CK18+ cells was apparent (shown in Fig 5D and 5E). In contrast, cells within the epithelial compartments of the abnormally structured proximal ductal regions in both 12Gso/*Tbx18*^*LacZ*^ (Fig G,H) and PB-Cre4^*+*^;*Tbx18*^*flox/flox*^ mutants (Fig 5J and 5K) displayed significantly reduced expression of both epithelial markers.

We also found that the distribution of SMA and VIM-positive cells was very similar in the normally structured distal regions of mutant anterior prostates and the prostates of wild type littermates. Specifically, VIM+ cells were distributed around the outside of the normally structured ducts in mutant mice, and VIM staining did not co-occur with SMA (Fig 5F). In contrast, in the disorganized proximal regions of the mutant anterior prostates we observed clusters of stromal cells co-staining with VIM and SMA (arrows in Fig 5I and 5L). This pattern was observed in multiple sections across proximal regions of the anterior prostate in both types of *Tbx18* mutants (not shown). In addition, we observed unusually large numbers of cells marked by VIM but not with SMA, in the periductal space surrounding the myofibroblastic cells and the abnormal epithelium in the mutant anterior prostate lobes. This staining pattern suggests an abnormal increase in numbers and organization of stromal fibroblasts in the mutant prostates.

### Significant gene expression differences presage *Tbx18* mutant prostate pathology

The subtle UGS phenotype observed in newborn animals (Fig 3), in conjunction with the fact that *Tbx18* transcription was first detected at significant levels in UGS at E16.5 (Fig 1), indicated that the earliest detectable molecular changes associated with *Tbx18* deficiency would be discovered between E16.5 and P0. This time period is also critical for the formation of the first prostate buds and marks the beginning of mesenchymal condensation around the nascent prostate buds [7,45]. To determine potential gene expression changes in *Tbx18*-null UGS, we collected whole UGS from E16.5 and E18.5 *Tbx18*^*GFP/GFP*^ mutant male embryos and littermate wild type controls. For each stage, we isolated RNA and performed RNA-Seq, comparing gene expression in mRNA from pooled tissues from each genotype at each stage. Gene expression analysis identified 324 differentially expressed genes (DEGs) at E16.5 and 6101 DEGs at E18.5 with an absolute fold change greater than or equal to 2.0; including genes with absolute fold change above 1.5 at the earlier stage identified a total of 694, many of which were further dysregulated at the later stage (S1 Table). To determine which major functional categories were differentially expressed at these two stages, we performed Gene Ontology (GO) analysis on the DEG sets using the DAVID functional analysis program (Table 1) [46].

**Table 1:** Functional categories enriched in RNA-seq comparison of E16.5 and E18.5 *Tbx18* mutant compared to wild type UGS.

	Enriched Functional Categories	
	E16.5	E18.5
**Down-Regulated DEGs**	Myofibril	Regulation of transcription
	Muscle organ development	Zinc finger / zinc ion binding
	Muscle system process	Chromosome organization
	Muscle cell differentiation	mRNA processing
	Sarcoplasmic reticulum	Microtubule organizing center
	Cytoskeletal protein binding	Cell cycle
	Myosin complex	Ribonucleoprotein complex
	Cardiac muscle tissue development	Cellular response to stress
	Myogenesis	Programmed cell death
		Establishment of protein localization
		Regulation of mesenchymal cell proliferation
		Regulation of epithelial cell proliferation
		Prostate gland growth
		Skeletal system development
		Serine threonine kinase activity
		Regulation of apoptosis
**Up-Regulated DEGs**	Peptidase inhibitor activity	Ribosomal protein / ribosome
	Extracellular region	Mitochondrion
	Defense response to bacterium	ncRNA metabolism
	Complement and coagulation cascades	Proteinaceous extracellular matrix
	Nucleosome assembly	Response to nutrient levels
	High density lipoprotein particle	Wnt superfamily
		NF-KB binding
		Oxidative phosphorylation
		Regulation of smooth muscle proliferation
		Regulation of Ras GTPase activity

The functions of genes that were significantly up-regulated or down-regulated in E16.5 and E18.5 *Tbx18*^*GFP/GFP*^ compared to wild type urogenital sinus (S1 Table) were analyzed separately using the DAVID functional clustering algorithm [46]. The leading GO term in significantly enriched functional clusters are shown in order of their enrichment.

At E16.5 nearly every enriched Functional Category was related to myogenesis and muscle structure. The most highly enriched of these categories included genes that encode structural components of myofibrils including Smooth muscle actins (*Acta2*, *Actg2*), Calponin 1 (*Cnn1)*, smooth muscle Myosin heavy chain 11 (*Myh11*), and Transgelin (*Tagln*) all of which were significantly down-regulated in mutants at E16.5. Genes encoding protease inhibitors (particularly the Stephin family of Cathepsin inhibitors; *Stfa1*, *Stfa2*, *Stfa2l1*, *Stfa3*), extracellular components (*Col10a1*, *Gdf15*, *Fgg*, and *Fgb*) and genes involved in defense response, the complement cascade, and inflammatory response (e.g. *S100a8*, *S100a9*, *C3*, *Cd52*, *Masp2*) were significantly up-regulated at this early stage (Table 1).

Despite the fact that major morphological differences were not observed in mutant UGS until P0, RNA-seq analysis of E18.5 mutant UGS detected a massive difference in gene expression between mutant and wild type mice with highly significant functional enrichments. Most notably, down-regulated DEGs were enriched for transcription factors including the key muscle differentiation factors including *Mef2a*, *Mef2c*, and *Mef2d*. At this stage, we also observed significant differential expression of TF genes including *Sox9*, *Hoxd13*, *Pten* and *Trp63*, all genes with important roles in prostate development and pathologies [17,26,47,48]. Genes regulating apoptosis and the proliferation of mesenchymal and epithelial cells were also down-regulated (Table 1); here we should note that because of its action in other tissues, *Tbx18* itself is annotated in both the apoptosis and cell proliferation categories, as are down-regulated DEGs *Shh*, *Trp63* and *Fgfr2*.

Tellingly, components of the extracellular matrix including eight Collagen, nine Matrix metalloprotease, as well as *Wnt* genes were also significantly up-regulated (Table 1), all of which have known roles in myofibroblast formation. DEGs associated with regulation of smooth muscle proliferation (including *Bmp4*, *Notch4*) were also enriched in the up-regulated genes. We also observed at this stage a massive up-regulation of genes directly involved in the formation of the ribosomes and mitochondrial components which are also up-regulated in myofibroblasts [49]. To confirm the results of the RNA-Seq study, we validated several genes at both developmental time points. Using RT-qPCR on RNA from individual UGS samples, we found that the results were highly consistent with the differential expression values from the pooled tissues used in sequencing (S1 Fig).

To assign particular genes and functions to mesenchymal or epithelial compartments, we cross-referenced our DEG sets with genes enriched in dissected UGS-M or UGS-E in a published microarray study (overlaps marked as M or E in S2 Table) [34]. Through this intersection, E16.5 functions related to regulation of myogenesis, muscle structural proteins, and inflammation were assigned to UGS-M; at E18.5 functions related to mitosis, mesenchymal cell development, and negative regulators of apoptosis were significantly down-regulated, while the C1Q complement system, regulation of cell shape, and *Hedgehog* signaling were up-regulated in the mesenchymal compartment. At the later stage, functions related to epithelial cell development, apoptosis, apical junctions, and prostate gland development were assigned to the UGS-E, while up-regulated functions related to cell adhesion and cell migration, NADP metabolism, secretion, and *Wnt* superfamily members were also associated with in the epithelial compartment (S2 Table).

## Discussion

This study provides the first evidence that *Tbx18* is required for normal differentiation of the prostatic stroma and that it indirectly influences the organization, proliferative control, and cellular identity of prostate epithelium. Using three different mutant genotypes, we show that *Tbx18* loss-of-function in the UGS during late embryogenesis and early postnatal life results in grossly disorganized stromal and epithelial layers of the anterior prostate in young adults, with many of the periductal stromal cells displaying a myofibroblastic phenotype.

Analysis of gene expression in mutant embryos revealed a cohort of functionally related, differentially expressed genes that may presage this later pathology. Most notably, genes involved in muscle development were already significantly down-regulated, while genes associated with extracellular matrix remodeling and inflammatory response were up-regulated in mutants as early as E16.5. These up-regulated functions are the same as those known to be associated with the appearance of myofibroblasts and epithelial dysplasia in adults [30], indicating a predisposition for the formation of later pathologies at this very early age. Since E16.5 is also the time when *Tbx18* is first robustly detected in wild type prostates, the data suggest an essential role for *Tbx18* in the regulatory hierarchy controlling expression of those genes.

By P0, we observed the first signs of morphological differences in developing mutant prostates including decreased density of UGS-M cells surrounding the prostate buds, possibly linked to proliferative failure of *Tbx18-*deficient mesenchymal cells as observed for ureter development [3]. In addition to these stromal abnormalities, the epithelium displayed increased thickness and subtle loss of organization in *Tbx18* mutants at this stage. However, these minor morphological changes belie the significant molecular and genetic changes that have already taken place within these cells.

Prominent among down-regulated DEGs in E18.5 embryos were transcription factors and signaling pathways with important roles in mediating cell-type specific differentiation in prostate. For example *Hoxd13* loss-of-function mutations result in reduced anterior prostate size with increased mesenchymal thickness [50]. *Foxa1* directly promotes cell growth in the prostate via an *AR*-dependent pathway and indirectly regulates other genes that are essential to prostate development, including *Shh* and *Bmp4* [51] which were also dysregulated in *Tbx18*^*GFP/GFP*^ UGS at E18.5. *Sox9*, which interacts with *Tbx18* in ureter development [52], was also significantly dysregulated at E18.5; this gene is already well established for its critical role in prostate development. For example, inactivation of *Sox9* before Androgen Receptor activation results in complete agenesis of the prostate [47,53]; at later developmental stages, *Sox9* is required to promote basal epithelial proliferation and is regulated by *Wnt* signaling through β-catenin [54,55]. Interestingly, many of these same developmental regulators are dysregulated in prostate cancer [23,47,51,55], and their early mis-expression may be linked to the cellular changes we detected in young adult mutant mice.

Although the phenotype of newborn mutant animals does not reflect the massive shift in the prostate gene expression that is already evident at E18.5, the failure of developmental and signaling pathways and the signature of inflammation are manifested clearly in 5 week-old mice. In contrast to the ureter, where the epithelium is reduced in thickness and cell numbers as well as organization in *Tbx18*^*-/-*^ mice [2,56], the cells that populate the adult prostate epithelium are not obviously reduced in number and instead, the numbers of cells in the epithelial compartment appear to have increased. These differences may stem from the distinct developmental origins of the epithelial layers and the paracrine factors they produce, as ureter epithelium is of mesodermal origin, and UGS-E is endodermal.

However, these differences in epithelial phenotype may also reflect different fates of *Tbx18-*deficient stroma in ureter and prostate. In particular, whereas *Tbx18-*deficient cells in ureter undergo apoptosis and are replaced by a thin layer of fibroblasts (Airik 2006), in prostate these cells appear to be replaced by a myofibroblasts (MFBs), which support the proliferation of epithelial cells [57]. The origins of these MFBs and the role of *Tbx18* in their formation are still unclear and will require an in-depth developmental study. MFBs are most commonly thought to differentiate from fibroblasts, and it is possible that *Tbx18-*prospective cells are first replaced by fibroblasts that further differentiate to MFBs in the prostate environment. We did detect an increase of Vimentin-positive cells in the lumen of mutant glands, and these might serve as a reservoir for MFBs.

However, other possibilities should be considered. For example, Vimentin is expressed widely throughout the prostate stroma during prenatal development, and VIM+SMA+ MFBs are also present during these stages, presumably reflecting an intermediate differentiation state [58,59]. Therefore, one possibility is that in the absence of *Tbx18*, mesenchymal cells destined for the SMC fate may simply fail to differentiate beyond this transitional stage. Alternatively, MFBs can also arise directly from mesenchymal stem cells that reside in human prostate, a process that is inhibited by the TGFB1-regulated TF, *RUNX1* [60]. *Runx1* was significantly down-regulated in *Tbx18-*mutant UGS at E18.5, raising the possibility that *Tbx18-*deficient mesenchymal precursors are redirected in mutant prostate anterior lobes to differentiate into MFBs. Using the *Tbx18*^*flox*^ allele in combination with PB-Cre4 and other mesenchymally-expressed *Cre* alleles, these possibilities can now be tested.

Whatever their origins, the appearance of MFBs in the *Tbx18* mutant stroma provides a potentially novel link between the developmental activities of this T-box factor and predisposition to adult prostate disease. MFBs are associated with multiple types of human prostate pathologies, including prostate cancer as well as benign prostate hypoplasia, and prostatic fibrosis [30,57,61]. Most of the factors that have been linked to MFB differentiation are expressed in adult prostate, either normally or in response to injury, infection, or other inflammatory events. However, as far as we can discern from RT-qPCR, IHC, and lineage tracing studies, *Tbx18* is expressed only transiently during early development and is not reactivated in the prostates of either healthy or mutant adults. These data suggest that a deficiency of *Tbx18* during a limited period of perinatal development is sufficient to predispose animals to frank prostate disease later in life. We speculate that the loss of *Tbx18* sets the stage for later prostate dysfunction through loss of a functional SMC stroma, but also due to the early creation of an inflammatory stromal environment that supports differentiation of MFBs.

Finally, from these data it remains unclear which stromal factors are key to the abnormalities in epithelial proliferation, differentiation and organization we observed since a large number of pathways predicted to influence these processes were dysregulated together in *Tbx18* mutant mice. Nevertheless, it is clear from our observation of these phenotypes in several different *Tbx18* genotypes that these factors are under the control, either directly or downstream and indirectly, of *Tbx18* activity. Future studies will focus on pinpointing the mechanisms of *Tbx18* activities in prostate development, and examine in depth the long-term effects of *Tbx18* insufficiency during the earliest postnatal stages on prostate health in later adult life.

## Supporting Information

S1 Fig Validation of differentially expressed genes. *In the RNA-Seq experiment we* analyzed pools of tissues from three individuals for each genotype and each stage. To validate that the pooled averages reflect individual samples we performed RT-qPCR on individual samples. The Fold Changes observed in RNA-Seq (no error bars) are presented next to the FCs obtained from qPCR on individual animals, with error bars showing the degree of variation between the three samples.
(TIFF)

S1 Table E16.5 and E18.5 differentially expressed genes. Differentially expressed genes from comparisons of mRNA from whole urogenital sinus (UGS) dissected from *Tbx18*^*Gfp/Gfp*^ null embryos and wild-type littermates at E16.5 and E18.5, respectively (as designated in column A). Column B indicates genes assigned as being enriched in mesenchymal (M) or epithelial (E) UGS compartments by Blum and colleagues [34]; only genes that were assigned unambiguously in that study are marked. Log2 fold change (column L) and other data columns were taken directly from the output of CuffDiff analysis, as described in Methods.
(XLSX)

S2 Table Functional categories enriched in DEGs assigned to UGS mesenchymal or epithelial compartments. DEGs marked as M (for UGS-M) or E (UGS-E) in S1 Table were analyzed with the DAVID Functional Annotation Clustering algorithm. Up- and Down-regulated DEGS were analyzed separately. Clusters of categories are represented here by the most highly enriched category in the cluster. Only functional category clusters with DAVID Enrichment Score >1.3 [thus with a geometric mean of combined *P* values ≥ 0.05, (46)] are listed, in order of enrichment for each class of genes.
(DOCX)

S3 Table List of RT-qPCR primers used for validation of differential gene expression. The primer sets listed were used to validate expression in individual E16.5 and E18.5 animals comprising a sequenced pool, with results reported in S1 Fig. Primer sets are named after the target gene, with “-f “after each gene name denoting “forward”, and “-r” denoting “reverse” primer designs.
(XLSX)
