# Correction: *Tbx18* Regulates the Differentiation of Periductal Smooth Muscle Stroma and the Maintenance of Epithelial Integrity in the Prostate

**DOI:** 10.1371/journal.pone.0157283

**Published:** 2016-06-03

**Authors:** The PLOS ONE Staff

In Fig 2, the labels for the individual stains are missing. Please see the corrected Fig 2 here. The publisher apologizes for the error.

**Fig 2:**
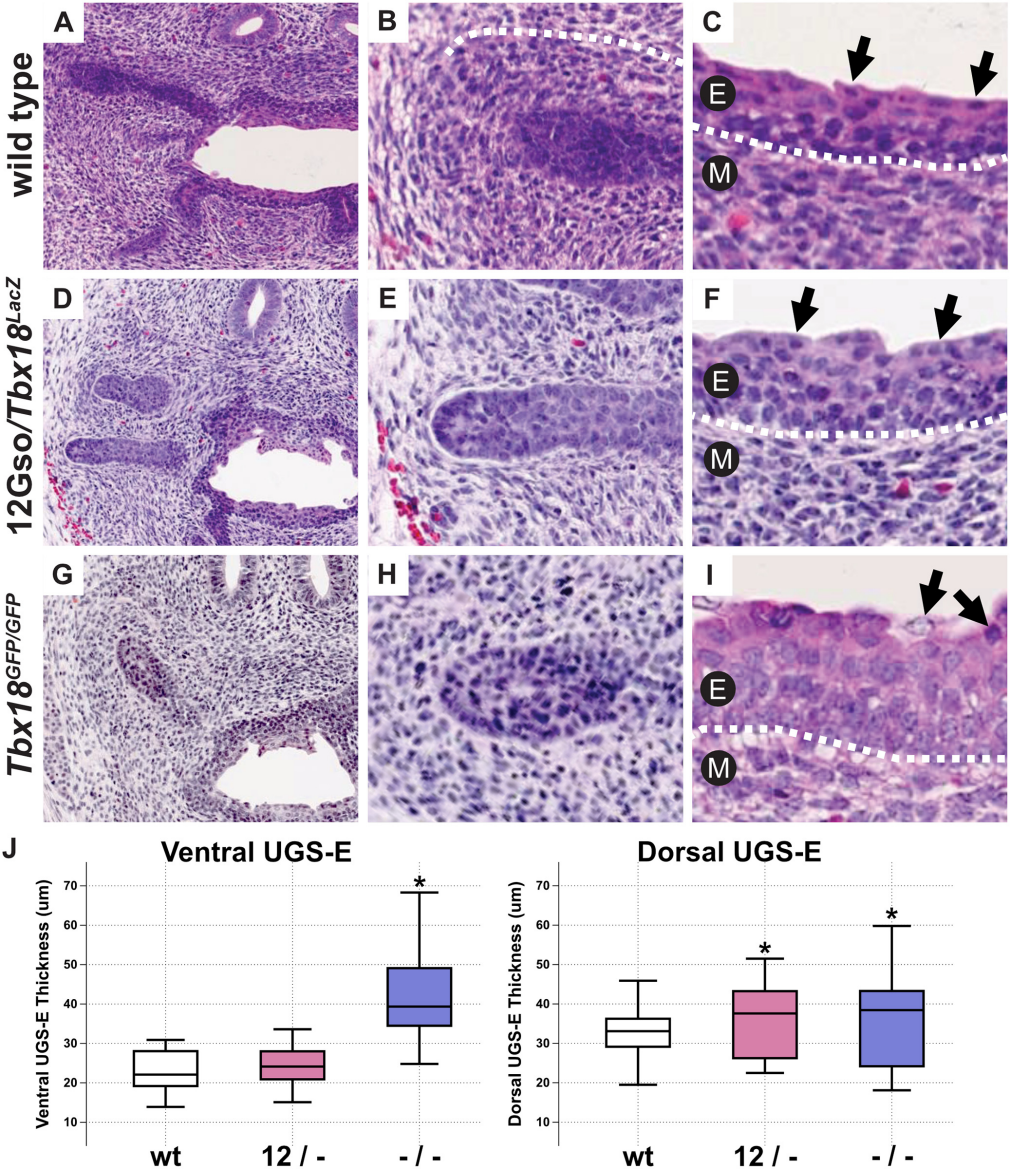
*Tbx18* LOF phenotype in P0 UGS. (A-I) H&E stains of P0 urogenital sinuses. (A-C) Wild type histology shows high cell density in the mesenchyme surrounding the epithelial prostate buds (A, B), and urethral epithelium is composed of 4–6 cell layers with a smooth apical surface (C). (D-F) 12Gso/*Tbx18*^*LacZ*^ compound heterozygotes present an intermediate phenotype in the UGS mesenchyme and the urethral epithelium. (G-I) *Tbx18*^*GFP/GFP*^ mutants have very low mesenchymal cell density surrounding epithelial prostate buds. The urethral epithelium in these mutants is increased in thickness with larger cell volumes (compare arrows in C, F, and I). (J) Measurements of the epithelial thickness in the urethral epithelium. The epithelium on the dorsal side is significantly increased in thickness compared to wild type littermates.

## References

[1] Bolt CC, Negi S, Guimarães-Camboa N, Zhang H, Troy JM, Lu X, et al (2016) *Tbx18* Regulates the Differentiation of Periductal Smooth Muscle Stroma and the Maintenance of Epithelial Integrity in the Prostate. PLoS ONE 11(4): e0154413 doi: 10.1371/journal.pone.0154413 27120339 10.1371/journal.pone.0154413 PMC4847854

