# Supplementary figures and images for: Tbx18 Regulates the Differentiation of Periductal Smooth Muscle Stroma and the Maintenance of Epithelial Integrity in the Prostate
Source: PLoS One. 2016 Apr 27;11(4):e0154413. doi: 10.1371/journal.pone.0154413 (PMC4847854; doi:10.1371/journal.pone.0154413)

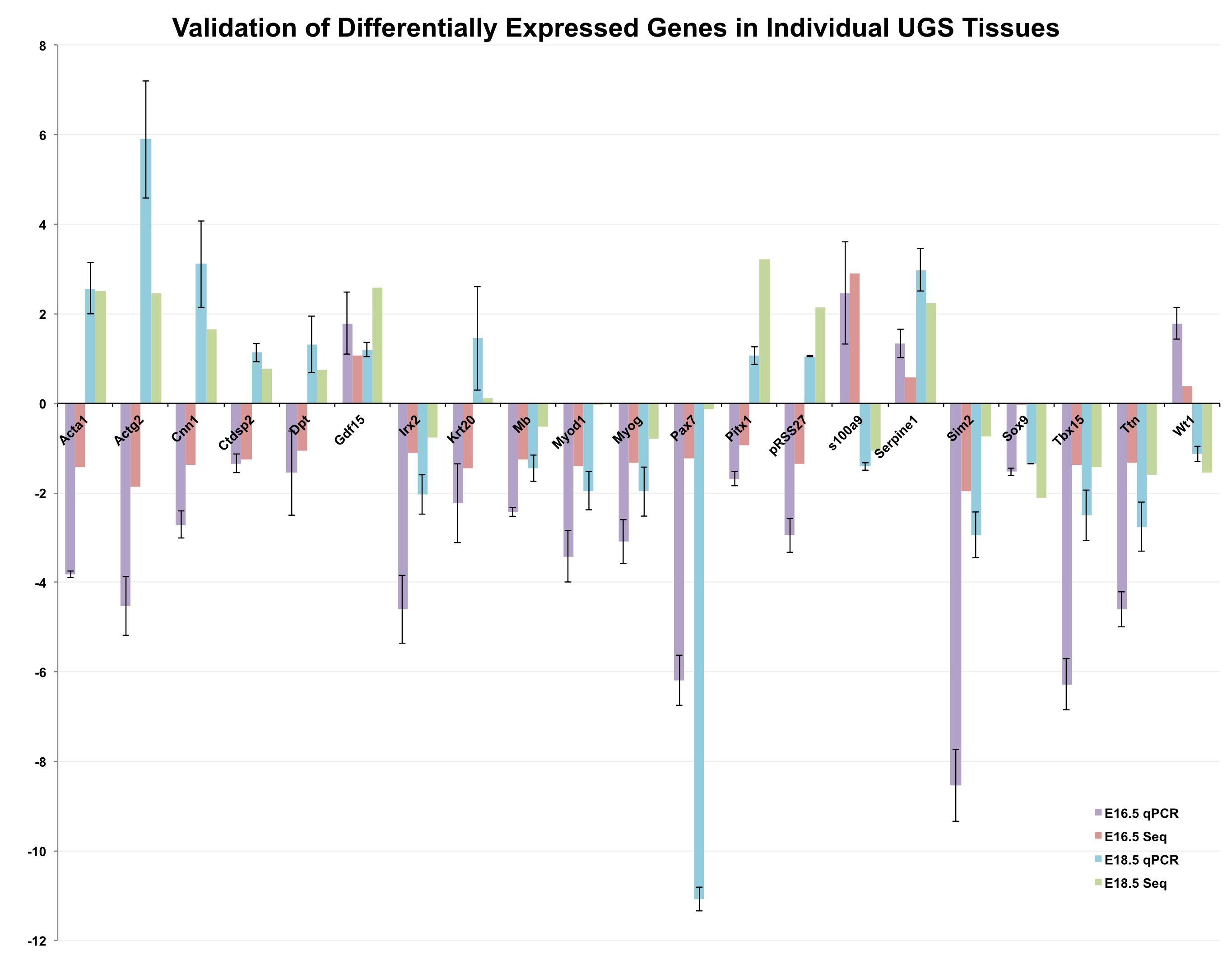

Supplement: S1 Fig — In the RNA-Seq experiment we analyzed pools of tissues from three individuals for each genotype and each stage. To validate that the pooled averages reflect individual samples we performed RT-qPCR on individual samples. The Fold Changes observed in RNA-Seq (no error bars) are presented next to the FCs obtained from qPCR on individual animals, with error bars showing the degree of variation between the three samples. (TIFF) [file pone.0154413.s001.tiff]
