# Supplementary material for: Tbx18 Regulates the Differentiation of Periductal Smooth Muscle Stroma and the Maintenance of Epithelial Integrity in the Prostate
Source: PLoS One. 2016 Apr 27;11(4):e0154413. doi: 10.1371/journal.pone.0154413 (PMC4847854; doi:10.1371/journal.pone.0154413)
Supplement: S2 Table — DEGs marked as M (for UGS-M) or E (UGS-E) in S1 Table were analyzed with the DAVID Functional Annotation Clustering algorithm. Up- and Down-regulated DEGS were analyzed separately. Clusters of categories are represented here by the most highly enriched category in the cluster. Only functional category clusters with DAVID Enrichment Score >1.3 [thus with a geometric mean of combined P values ≥ 0.05, (46)] are listed, in order of enrichment for each class of genes. (DOCX) [file pone.0154413.s003.docx]

| **S2 Table. Functional category clusters enriched in DEGS assigned to UGS-M or UGS-E** | | | | |
| --- | --- | --- | --- | --- |
|  | **E16 UGS-M (1.5FC)** | **E18 UGS-M** | **E16 UGS-E (1.5FC)** | **E18 UGS-E** |
| **Down-regulated** | Muscle protein  Cytoskeletal protein /actin binding  Intermediate filament  Structural constituent of cytoskeleton  Cardiac muscle development  Muscle cell differentiation  Actin cytoskeleton  Myogenesis  Calmodulin binding  Myosin complex  Regulation of cell migration  Muscle organ development  T-box transcription factor  Tissue morphogenesis  Sarcoplasm  Tube development | **Mitotic cell cycle**  Regulation of transcription  Zinc finger, C2H2 type  Ras GTPase  Microtubule cytoskeleton  Synaptic transmission  Blood vessel development^  Tube development  Coated vesicle  Cell morphogenesis involved in differentiation  Vesicle mediated transport  Regulation of mesenchymal cell proliferation  Synaptogenesis  Regulation of cell migration  Long term potentiation  Mesenchymal cell development  **Negative regulation of apoptosis**  Cell migration  Urogenital system development | Cytoskeletal protein/actin binding | Zinc finger, C2H2 type  Regulation of transcription  Cellular macromolecular catabolic process/protein catabolism  Chromosome organization  Activator/DNA binding  Apical Junction Process  Epithelial cell differentiation  **Apoptosis**  Cytoskeletal organization/actin filament organization  **Regulation of apoptosis**  Protein transport  Regulation of T-cell receptor signaling  Response to DNA damage stimulus  Prostate gland morphogenesis  Regulation of muscle development |
| **Up-regulated** | Signal/secreted  Defense response to gram positive bacteria | Extracellular region  Cytoskeletal protein/actin binding  Collagen  C1Q complement protein  Sarcomere  Regulation of cell shape  Blood vessel development^  Sarcoplasm  Muscle protein  Cell adhesion  Calponin-like actin binding  Negative regulation of cell migration  Hedgehog signaling pathway | (None significant) | Cytoskeletal keratin  Cell adhesion  Pyridoxal phosphate binding  Vesicle mediated transport  Secretion  NADP metabolism  Cell migration  Ligand dependent nuclear hormone receptor activity  WNT superfamily |

^ Up-regulated includes: Bmp4, Acvrl1, Notch4, Egfl7; Down-regulated: Fgf9, Tbfgbr1, Tgfb2, Pten, Rbpj
